# scKINETICS: inference of regulatory velocity with single-cell transcriptomics data

**DOI:** 10.1093/bioinformatics/btad267

**Published:** 2023-06-30

**Authors:** Cassandra Burdziak, Chujun Julia Zhao, Doron Haviv, Direna Alonso-Curbelo, Scott W Lowe, Dana Pe’er

**Affiliations:** Computational and Systems Biology Program, Memorial Sloan Kettering Cancer Center, Sloan Kettering Institute, 408 E 69th Street, New York, NY 10021, United States; Computational and Systems Biology Program, Memorial Sloan Kettering Cancer Center, Sloan Kettering Institute, 408 E 69th Street, New York, NY 10021, United States; Department of Biomedical Engineering, Columbia University, 1210 Amsterdam Ave, New York, NY 10027, United States; Computational and Systems Biology Program, Memorial Sloan Kettering Cancer Center, Sloan Kettering Institute, 408 E 69th Street, New York, NY 10021, United States; Institute for Research in Biomedicine (IRB Barcelona), The Barcelona Institute of Science and Technology (BIST), Carrer de Baldiri Reixac, 10, Barcelona 08028, Spain; Cancer Biology and Genetics Program, Memorial Sloan Kettering Cancer Center, Sloan Kettering Institute, 408 E 69th Street, New York, NY 10021, United States; Cancer Biology and Genetics Program, Memorial Sloan Kettering Cancer Center, Sloan Kettering Institute, 408 E 69th Street, New York, NY 10021, United States; Howard Hughes Medical Institute, 4000 Jones Bridge Road, Chevy Chase, Maryland 20815, United States; Computational and Systems Biology Program, Memorial Sloan Kettering Cancer Center, Sloan Kettering Institute, 408 E 69th Street, New York, NY 10021, United States; Howard Hughes Medical Institute, 4000 Jones Bridge Road, Chevy Chase, Maryland 20815, United States

## Abstract

**Motivation:**

Transcriptional dynamics are governed by the action of regulatory proteins and are fundamental to systems ranging from normal development to disease. RNA velocity methods for tracking phenotypic dynamics ignore information on the regulatory drivers of gene expression variability through time.

**Results:**

We introduce *scKINETICS* (Key regulatory Interaction NETwork for Inferring Cell Speed), a dynamical model of gene expression change which is fit with the simultaneous learning of per-cell transcriptional velocities and a governing gene regulatory network. Fitting is accomplished through an expectation–maximization approach designed to learn the impact of each regulator on its target genes, leveraging biologically motivated priors from epigenetic data, gene–gene coexpression, and constraints on cells’ future states imposed by the phenotypic manifold. Applying this approach to an acute pancreatitis dataset recapitulates a well-studied axis of acinar-to-ductal transdifferentiation whilst proposing novel regulators of this process, including factors with previously appreciated roles in driving pancreatic tumorigenesis. In benchmarking experiments, we show that *scKINETICS* successfully extends and improves existing velocity approaches to generate interpretable, mechanistic models of gene regulatory dynamics.

**Availability and implementation:**

All python code and an accompanying Jupyter notebook with demonstrations are available at http://github.com/dpeerlab/scKINETICS.

## 1 Introduction

Advances in single-cell genomics have uncovered vast cellular heterogeneity during organismal development, regeneration, and in disease states. The emergence and maintenance of this phenotypic diversity is largely owed to the combined action of a handful of transcription factors (TFs), which bind to regulatory regions of the genome and drive activation or repression of each expressed gene. As TFs are in turn subject to their own regulation, dynamic changes to cellular phenotype can be enacted by modifying TF activity in response to cell-intrinsic or cell-extrinsic stimuli. Hence, mechanistic insights into both static and dynamic heterogeneity can be derived from the study of differential TF activity over time and phenotypic space.

Numerous approaches have been devised to measure regulatory activity from single-cell data, often by inferring gene regulatory networks (GRNs) associating TFs and the genes they regulate (target genes). Whether these links are inferred from gene expression (Aibar et al. 2017), epigenetic data (Dong et al. 2022), or a combination of both (Jansen et al. 2019), many such methods consider fixed clusters of cells representing the static endpoints of differentiation. We recently introduced *Symphony*, a Bayesian approach which models single cells in this manner as a mixture of individual cell-type-specific programs defined by unique GRNs (Bachireddy et al. 2021). In contrast, studies of transcriptional dynamics avoid fixed cell type definitions, and instead apply trajectory inference to order cells along an axis from the most immature to mature populations. This may rely on prior knowledge of the cellular progenitors of downstream lineages (Setty et al. 2016, 2019) or, more recently, on unbiased inference to orient each cell’s future state from its current expression. The latter class of methods typically defines the rate of change (or *velocity*) of a cell’s expression, assuming a causal role of features in time-oriented processes of RNA splicing (La Manno et al. 2018) or chromatin remodeling (Li et al. 2023).

Explicitly, velocity algorithms fit a system of ordinary differential equations (ODEs) for each gene, modeling causal factors (e.g. unspliced RNA) whose current values influence the rate of change of downstream factors (e.g. spliced RNA). Inferring parameters of this system allows for the extrapolation of (un)spliced expression to the future (t>0), given the initial state of each cell (t=0). Much effort has been expended on improving these methods to combat noisy measurements of individual features (Lange et al. 2022), improve parameter inference (Bergen et al. 2020), and encode nonlinearity in the model for gene expression changes (Chen et al. 2022).

Velocity approaches may now accurately trace dynamic changes with limited requirements for prior knowledge, yet the inference of regulatory mechanisms accompanying such changes remains underdeveloped. A recent approach, *MultiVelo* (Li et al. 2023), takes a first step by incorporating epigenetic measurements into the velocity framework. Using multiomic data measuring both RNA and chromatin accessibility in each cell, *MultiVelo* expands the ODE model to include the influence of chromatin opening and closing over time on the expression of nascent unspliced transcripts. This approach, like its predecessors, relies on sparse, noisy signal (from accessible chromatin in this case), which is often heavily undersampled compared with transcriptomic data. Most problematically, *MultiVelo* makes strong simplifying assumptions on the causality of regulatory dynamics, expecting that chromatin accessibility changes necessarily drive changes in expression.

In reality, gene regulation occurs as part of a complex system, in which multiple confounding features, such as the presence of cofactors, dictate the influence of underlying chromatin accessibility on gene expression. Current velocity approaches, whether modeling dynamics in mRNA splicing or chromatin accessibility, fail to account for genome-wide patterns of regulatory change, which have yet to be considered for their likely preeminent role in influencing cell fate. In turn, approaches which do model dynamic regulatory networks do so without unbiased trajectory inference (e.g. *SCRIBE* by Qiu et al. 2020), and hence are limited to applications in well-studied regimes with known axes of differentiation. Ideally, regulation and its consequences on cell states can be *jointly* learned from single-cell data, which contain abundant information on both regulator and target activities (via their expression) and cell-state evolution through time (via the phenotypic manifold).

## 2 Approach

Here, we propose *scKINETICS*, an integrative algorithm which combines inference of regulatory network structure with robust *de novo* estimation of gene expression velocity under a model of causal, regulation-driven dynamics. *scKINETICS* first utilizes epigenetics as prior information to inform the regulators driving the change in expression of each gene. Unlike previous approaches which treat the dynamics for each gene independently (La Manno et al. 2018; Bergen et al. 2020; Li et al. 2023), we model changes in cellular phenotype with a joint system of dynamic equations governing the expression of each gene as dictated by these regulators within a genome-wide GRN. An expectation–maximization (EM) approach iteratively learns the precise TF influence per-gene and velocities per-cell, jointly leveraging epigenetic data and coexpression patterns. We further incorporate higher order phenotypic features (e.g. cell–cell similarity) to seed velocities in an appropriate direction given the observed phenotypic manifold. In this way, the velocities derived from *scKINETICS* are often more accurate than approaches relying on weaker or noisier signals, and further provide insights into the precise regulators underlying phenotypic change without strong requirements for prior knowledge.

## 3 Materials and methods

As with previous velocity algorithms, the core of *scKINETICS* is an ODE model of the genes’ expression through time. We adopt a system of linear, time-homogeneous differential equations, with one function $x_{i}^{\prime }(t)$ describing the rate of change for an individual gene *i*. Our system thus has dimensionality equal to the number of measured genes; this is in contrast to previous methods, which establish a separate system for each individual gene to produce independent velocity estimates per gene.

**Figure 1.**
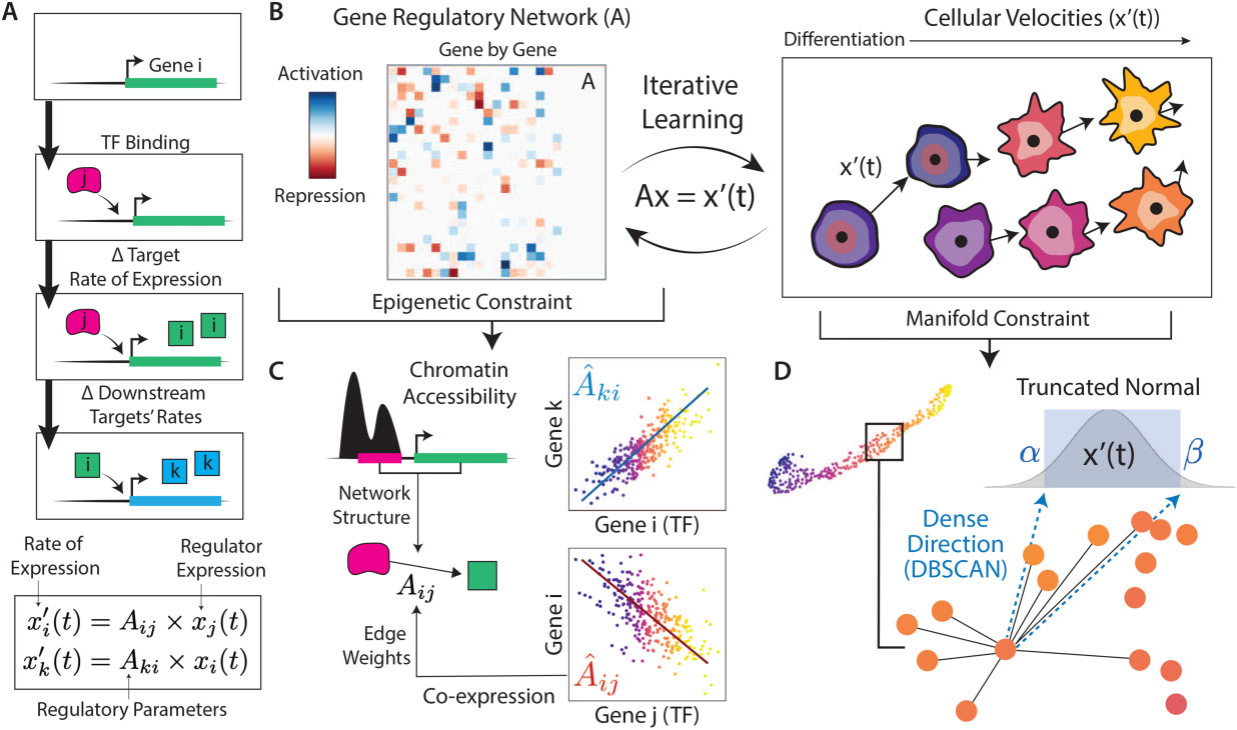
*scKINETICS* algorithm for joint inference of gene regulatory mechanism and cellular velocity. (A) The expression rate of each target gene is a function of the expression level of the TFs which bind to the genome near its transcription start site. This interaction can be summarized as a differential equation, where a regulatory parameter is indexed by the TF–target pair and indicates the strength of the regulatory impact. When TFs are in turn subject to regulation (TF *i*), this defines a system of equations dictating the expression rate of each regulated gene. (B) The iterative learning process of *scKINETCS*. A cell-type-specific GRN (left), in which each element represents the impact of the regulating TF (columns) on a given target gene (rows). Cellular velocities (right) are vectors describing the direction of phenotypic change, in gene expression space, from initiating populations (purple) towards mature populations (yellow). GRN and velocity values are iteratively estimated with the EM algorithm, subject to constraints from accessibility data (C) or directions on the observed manifold (D). (C) Possible TF–target interactions defining the GRN in (B) are determined from chromatin accessibility (ATAC-seq) data marking active regulatory elements proximal to target genes where TF binding occurs. Edge weights (defining regulatory parameters $A_{ij}$), representing the interaction strength and direction (activation or repression), are based on a prior from coexpression magnitudes of each TF–target pair in scRNA-seq data. (D) Per-gene velocities are modeled as a truncated Gaussian distribution with lower and upper constraints determined as vectors spanning dense directions in the local neighborhood around a cell, where many cells are situated along the manifold.

The basic assumption of *scKINETICS* is that the rate of each gene’s expression is dictated by the expression level of each of its regulatory TFs at that particular point in time. Mathematically, we encode this assumption in the differential equation for target gene *i* with TFs $j=1\ldots d_{i}$ as follows: where $x_{j}$ represents the expression of TF *j* and *t* represents time. Each equation contains TF-specific rate parameters $A_{ij}$, which represent the impact of expression of the TF *j* on expression of the target *i*. These can take on positive values (denoting target activation) or negative values (denoting target repression) (Fig. 1A).


$$x_{i}^{\prime }(t)=\sum_{j=1}^{d_{i}}x_{j}(t)A_{ij}$$

Considering all genes, we can write this as the following linear system:


$${\vec{x}}^{\prime }(t)=A\vec{x}(t)$$

Here, $\vec{x}$ is the vector of expression for all genes, both targets and TFs; *A* is a matrix of rates, where $A_{ij}$ describes the effect of expression of TF *j* on target *i*. By design, $A_{ij}$ = 0 if gene *j* is not a regulator of gene *i*. Thus, *A* can be conceived as a GRN whereby the expression of targets is driven by its regulatory TFs, with magnitude and sign (activation or repression) of the interaction given by the values $A_{ij}$.

Of note, the system of equations can be solved analytically:


$$\vec{x}(t)=e^{At}\vec{x}(0)$$

Given this solution, we may extrapolate the expression patterns of target genes to future time points (t>0) based solely on the expression of regulators at the current or *starting* [t=0, i.e. x(0)] time point (Fig. 1A).

To perform velocity inference with *scKINETICS* requires knowledge only of the parameters in *A*, which is highly related to a GRN where nodes represent single genes and directed links between nodes represent direct regulation of the target gene by its regulators. Edges may be weighted by the strength of impact on the target gene, and also may be signed by the directionality (activation versus repression) of the interaction. The inference problem for *A* is identical to GRN inference, with edge magnitudes specifically representing the impact of each TF’s binding on the *rate* of target expression. Below, we detail our procedures for inferring network structure (Section 3.1) and learning edge weights and signs (Section 3.2).

### 3.1 Network structure estimation

The most basic feature of a GRN is its graph structure, representing which TFs impact the expression of—i.e. regulate—which target genes. Here, we use epigenetic data to constrain candidate TFs to those that potentially bind to promoters and enhancers proximal to the target gene. As in previous approaches (Bachireddy et al. 2021), candidate TF regulators can be derived directly from epigenetic data (e.g. ATAC-seq) by identifying canonical TF binding motifs in open chromatin regions near target genes. We use bulk or scATAC-seq data to call peaks in potential regulatory regions and scan the DNA sequence in each peak against a database of predetermined TF binding motifs (Weirauch et al. 2014) to associate each peak with potential TFs (Korhonen et al. 2009). TF-bound peaks are then mapped to putative target genes based on genomic distance (Yu et al. 2015) creating a mask of candidate TFs for each target gene: each TF *j* which binds in ATAC-seq peaks proximal to target gene *i* will enable a nonzero value for element $A_{ij}$, where all other elements in *A* will be constrained to 0. In other words, each target’s rate of change can only be positively or negatively influenced by TFs that bind in proximal regions (Fig. 1B). The final GRN structure incorporates additional information (e.g. coexpression, described in detail below) to further narrow the list of candidate TF–target pairs, ultimately producing a sparse network structure.

### 3.2 Parameter estimation

While approaches for determining a candidate GRN structure from epigenetic data are well established, inferring the precise impact that each TF exerts (i.e. the values for each nonzero element $A_{ij}$) is more challenging. As in previous approaches (Bachireddy et al. 2021), TF–target coexpression patterns (e.g. from scRNA-seq data) can indicate both the strength (or *magnitude*) and the direction (or *sign*) of a TF’s impact. However, coexpression may be confounded by multiple factors which produce high coexpression amongst noninteracting genes. The goal of GRN parameter estimation is thus to learn the optimal regulatory weights from these data representing *bona fide* TF–target interactions.

The learning problem boils down to estimating values of the parameter *A* which satisfy the model ${\vec{x}}^{\prime }(t)=A\vec{x}(t)$, where *A* has been masked by epigenetic data as described in Section 3.1. If velocities ${\vec{x}}^{\prime }(t)$ were observed, *A* could be fit with an ordinary least squares solution akin to any simple linear regression. Of course, we do not directly observe cellular velocities; hence, the problem is underdetermined, and a simple maximum likelihood solution cannot be used without additional data or constraints. The EM algorithm, which iteratively updates parameter estimates and latent variable values, is useful for such inference cases involving both observed ($\vec{x}(t)$) and unobserved (${\vec{x}}^{\prime }(t)$) variables. We thus derive EM updates below to iteratively solve for *A* and the velocities ${\vec{x}}^{\prime }(t)$, subject to biologically motivated constraints on each to avoid nonidentifiability (Fig. 1B).

#### 3.2.1 EM algorithm

The EM algorithm seeks to find a maximum likelihood estimate (MLE) for parameters whilst marginalizing out latent variables, thereby eliminating dependency on these unknowns during the maximization step. We infer the MLE for *A* by iteratively calculating an expectation of the data likelihood, marginalizing out velocities ${\vec{x}}^{\prime }(t)$ and updating *A* by taking a maximum over this expectation. This approach, inspired by previous EM applications to missing data (Park and Lee 2012), enables this by introducing additional constraints or data to permit inference in this underdetermined case. We introduce biologically motivated constraints on the values of our parameters (*A*) and latent variables (${\vec{x}}^{\prime }(t)$). Below we detail a prior on the former, which leverages TF–target coexpression patterns across cells to inform regulatory weights in *A* (the “Coexpression Prior”), and a constraint on the latter, which seeds the directionality of unknown velocity vectors along the phenotypic manifold (the “Manifold Constraint”).

##### 3.2.1.1 The coexpression prior

The key principle underlying our prior is that influences and interactions between biological entities generate statistical dependencies in the observed data (e.g. if TF *j* activates target *i*, then we expect to see high levels of *i* whenever levels of its activator *j* are high). This is the basis for many methods (Aibar et al. 2017; Chan et al. 2017) which infer regulatory strength based on coexpression patterns over bulk or single-cell measurements. We adopt a similar strategy here by assuming that the degree of coexpression between a TF *j* and a target *i* can serve as a loose prior on the weights $A_{ij}$. Explicitly, we populate a prior matrix $\hat{A}$ with the empirical covariance value for each interacting pair of genes. We then model *A* as follows: where $\hat{\sigma }$ is a user-defined parameter affecting the strength of the prior. Note that this design captures both magnitude and direction (activation versus repression) by allowing both positive and negative values of covariance to serve as priors (Fig. 1C). The coexpression matrix $\hat{A}$ serves as both a prior (as above) and an initial value for the parameters in the EM algorithm. As EM identifies a local optimum and is therefore sensitive to initialization, a biologically sensible starting value is critical for its success. Coexpression information is thus a major contributing component to the output of *scKINETICS*.


$$A_{ij}∼N({\hat{A}}_{ij},\hat{\sigma })$$

##### 3.2.1.2 The manifold constraint

Existing velocity approaches establish each cell’s future state entirely through the inference of parameters akin to *A*. This feature discards abundant information on the future state of each cell available in a dataset: indeed, for systems undergoing dynamic processes, we are likely to observe examples of each cell’s future state amongst its immediate neighbors. In similar vein to *CellRank* (Lange et al. 2022), we take advantage of this fact by including such neighborhood information in the constraints for latent velocities of each cell. However, the details of our approach are quite distinct: we assume that cells undergoing differentiation are programmed towards likely fates, and hence will give rise to immediate future states that will be both highly similar to their predecessors (i.e. *neighbors*) and reproducible (i.e. dense in phenotypic space). We capture the former by constructing a k-nearest neighbor (kNN) graph representing the phenotypic manifold. We assume that a subset of a cell’s neighbors may reflect a reproducible future state. In other words, a *cluster* of neighbors that share similar directions relative to the initial cell may be used to seed velocity vectors forward in time.

We apply *DBSCAN* (Ester et al. 1996) to identify a density of cells based on their relative angle (cosine distance) to the initial cell. A cluster of cells representing such a high-density region may then be interpreted as potential velocities, and hence will impose a probabilistic constraint on inferred velocities by applying the following model: where ${\vec{\alpha }}_{n}$ and ${\vec{\beta }}_{n}$ represent the extreme directions at the edges of each density, and provide bounds of truncation for the velocity distribution. Here, *TruncN* refers to univariate truncated normal distributions evaluated for each element of the vectors independently. Explicitly, for a given cell *n*, we identify an initial direction from the central cell *s* in a *DBSCAN* cluster comprised of a set of cell *n’*s neighbors. We define $\vec{\epsilon }$, a vector containing a small window size for each gene defining the width of the bounds of truncation, chosen based on the variability of each gene’s expression observed across neighbors (0.1 SDs in the experiments in this article). We then set ${\vec{\alpha }}_{n}$ and ${\vec{\beta }}_{n}$ such that they span a small window above and below the expression in the central *DBSCAN* cell ${\vec{x}}_{s}$: ${\vec{\alpha }}_{n}={\vec{x}}_{s}-\vec{\epsilon },{\vec{\beta }}_{n}={\vec{x}}_{s}+\vec{\epsilon }$.


$${\vec{x}}_{n}^{\prime }(t)∼\mathrm{TruncN}(A{\vec{x}}_{n},{\vec{\alpha }}_{n},{\vec{\beta }}_{n},\sigma )$$

As *DBSCAN* may identify many such densities and therefore many potential constraints for each cell, we choose the density with the highest agreement with our Coexpression Prior. Mathematically, we assume a “best guess” direction *v* as $v=\hat{A}\vec{x}$. The central cell amongst each *DBSCAN* cluster is compared with *v* by cosine distance to choose the most likely future state based on the TF–target coexpression patterns (Fig. 1D).

Given these priors, we have a generative model for velocities across cells ${\vec{x}}^{\prime }(t)|A,\vec{x},\vec{\alpha },\vec{\beta }$ and for parameters $A|\hat{A}$:


$$\prod_{i,j\in A_{i,j}\neq 0}N(A_{ij};{\hat{A}}_{ij},\hat{\sigma })\prod_{n\in \text{cells}}Tr\mathrm{uncN}({\vec{x}}_{n}^{\prime }(t);A{\vec{x}}_{n},{\vec{\alpha }}_{n},{\vec{\beta }}_{n},\sigma )$$

We use these to derive the following objective function defining the **E step**: where log $L(A|{\vec{x}}^{\prime },\vec{x}$) is the log-likelihood of *A* under fixed observed and unobserved data, and $A_{k}$ represents the value of *A* in the *k*th iteration. Updates for *A* in the **M step** can be obtained as follows:


$$Q(A|A_{k})=E_{{\vec{x}}^{\prime }|\vec{x},A_{k}}[\text{log}\;L(A|{\vec{x}}^{\prime },\vec{x})]$$

$$A_{k+1}=\underset{A}{\text{argmax}}\;Q(A|A_{k})$$

Here, we present the resulting update function for ${\vec{a}}_{i}$ containing the weights for each regulator of gene *i*. In particular, ${\vec{a}}_{i}$ is a vector with dimension equal to the number of regulators (determined by the epigenetic mask) for target gene *i*. Given fixed constraints α and β for target gene *i* across all cells, we can write the update function as follows: with ${\vec{x}}_{n}$ representing expression of gene *i’*s regulators in cell *n*, $\alpha_{n}$ and $\beta_{n}$ representing scalar constraints for target *i* in cell *n*, $\hat{\vec{a_{i}}}$ representing priors for weights, and ϕ and Φ representing the Gaussian PDF and CDF, respectively.


$${\vec{a}}_{i}^{\left(k+1\right)}=-{\left(E_{1}^{T}+E_{1}\right)}^{-1}E_{2}$$

$$E_{1}=-\frac{1}{2{\hat{\sigma }}^{2}}I-\frac{1}{2\sigma^{2}}\sum_{n=1}^{N}{\vec{x}}_{n}{\vec{x}}_{n}^{T}$$

$$E_{2}=\frac{1}{{\hat{\sigma }}^{2}}\hat{\vec{a_{i}}}+\frac{1}{\sigma^{2}}\sum_{n=1}^{N}{\vec{x}}_{n}{\vec{a_{i}}}^{(k)T}{\vec{x}}_{n}-\frac{1}{\sigma }\sum_{n=1}^{N}{\vec{x}}_{n}(\frac{\phi (\beta_{n})-\phi (\alpha_{n})}{\Phi (\beta_{n})-\Phi (\alpha_{n})})$$

This algorithm is implemented as part of our python package available at http://github.com/dpeerlab/scKINETICS. The required input is a scRNA-seq count matrix in AnnData format and bulk ATAC-seq, scATAC-seq, or multiome-derived peaks. This package includes functionality for input file generation (e.g. differential peak selection, TF motif calling, peak-to-target association, GRN construction, etc.), parallelizable and tunable EM model training, customizable 2D velocity visualization as in (La Manno et al. 2018; Bergen et al. 2020), and end-to-end analysis. In addition, it outputs a cell-specific TF activity score derived from our velocity predictions (detailed below in Section 4.4). A demo Jupyter notebook contains a sample analysis generating the results below on pancreas regeneration, using the full functionality of the package.

## 4 Results

We demonstrated *scKINETICS* on a system of pancreatic epithelial regeneration. The exocrine pancreas, composed mainly of acinar and ductal cell types, can regenerate robustly (Zhou and Melton 2018). During an inflammatory event such as pancreatitis triggered by injury, mature acinar cells *trans*-differentiate into a duct-like phenotype. When cells harbor an oncogenic mutation (e.g. *KRAS* mutation), this normal acinar-to-ductal metaplasia (ADM) response is derailed and initiates a sequence of events that can ultimately lead to pancreatic ductal adenocarcinoma (Kopp et al. 2012), a highly lethal cancer for which few treatment options exist.

Recent work leveraging scRNA-seq data from the mouse pancreas undergoing chemically induced pancreatitis reveals that mutant *KRAS* hijacks normal regenerative cell state transitions for tumor promotion (Burdziak et al. 2022). Though we possess some knowledge of the ADM axis, much remains unknown regarding the regulatory mechanisms underlying normal pancreas regeneration, a pivotal prerequisite for understanding how they derail in cancer. Therefore, we sought to apply *scKINETICS* to probe both the underlying dynamics and epigenetic regulation that drive normal ADM in mice as a reference towards a better understanding of this process in the tumorigenic setting.

Here, we demonstrate that velocity vectors inferred with our approach can accurately capture ADM (Section 4.2) and infer potential regulatory mechanisms driving regeneration in a TF-wide perturbation screen in mouse using *scKINETICS* output (Section 4.4). The latter presents a unique and powerful feature of our design that alternative velocity methods (e.g. *MultiVelo*, Li et al. 2023) do not address.

### 4.1 Input generation and model fitting

**Figure 2.**
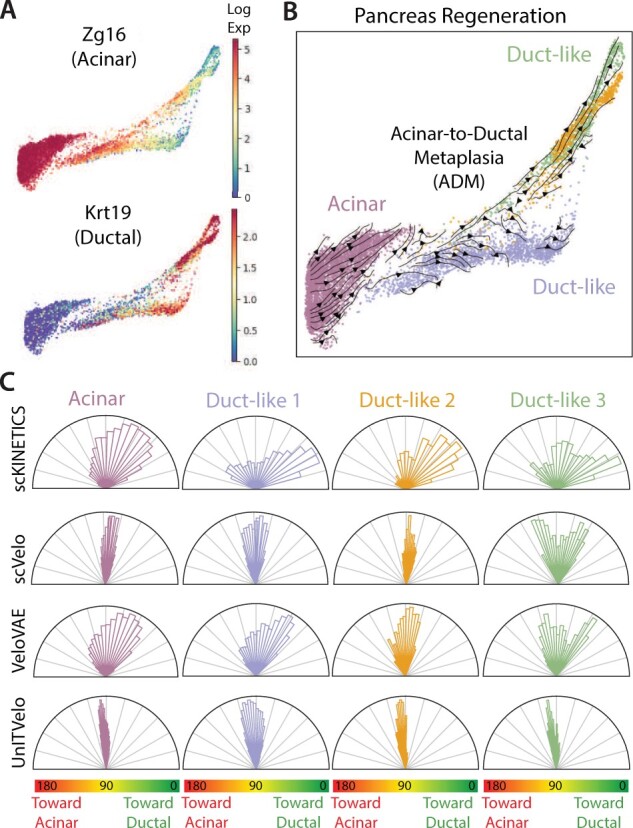
Inference of velocity in pancreatitis. (A) Logged expression of canonical acinar and ductal markers. Colorbar scaled from the 5th to 95th percentile values. (B) Velocity projection in two dimensions, characterizing ADM on a FDL embedding, as described in La Manno *et al.* (2018). Cells are colored by Phenograph cluster. (C) *scKINETICS* shows superior alignment to biological ground truth compared with competing algorithms. Rose plots show cosine distances between per-cell velocity vectors to the reference computed with pseudotime. 0°, 90°, and 180° denote angles toward ductal phenotypes, orthogonal, and toward acinar phenotypes, respectively. Each cluster (colored as in 2B) is plotted separately with 20 bins. Values closer to 0° (<90°) imply orientation of velocities along the ADM axis, those at 180° imply the opposite orientation, and orthogonal values imply lack of clear association to the ADM axis in either direction.

We obtained a processed scRNA-seq count matrix from Burdziak et al. (2022)  (GEO accession GSE207943), including cells from both normal and regenerating pancreas epithelia (5501 cells and 15497 expressed genes). Four major Phenograph clusters (Levine et al. 2015) reported in Burdziak et al. (2022) were manually annotated to acinar- or duct-like states based on the expression levels of canonical markers (e.g. *Zg16*, *Cpa1* for acinar and *Krt19*, *Sox9*, *Clu* for duct like). To emphasize the dynamics spanning these clusters, force-directed layout (FDL) coordinates were obtained with fa2 (Jacomy et al. 2014) on a kNN graph (*k* = 15) built on 50 principal components. As expected, we observed a continuum of states spanning acinar-like (*Zg16*${}^{+}$) and duct-like (*Krt19*${}^{+}$) extrema, with clear intermediate states harboring a mixed acinar and ductal phenotype (Fig. 2A).

In addition to scRNA-seq data, *scKINETICS* requires input epigenomic data to constrain potential regulatory interactions. Unlike *MultiVelo* (Li et al. 2023), which depends on multiome data measuring the paired epigenome and transcriptome of each cell, the flexible design of *scKINETICS* allows us to constrain velocities using epigenetic measurements at any resolution (e.g. multiome, scATAC-seq, or bulk ATAC-seq). In this case, we leveraged bulk ATAC-seq data collected from Alonso-Curbelo et al. (2021), representing an average accessibility profile across the four clusters of single cells. To then learn cell-type aware regulatory patterns, a separate regulatory model was fit for each of the clusters based on the same set of bulk-informed regulatory regions which are likely accessible in all four, but fit cluster-specific gene–gene covariance patterns that indicate different regulation.

In more detail, we obtained a set of regulatory elements (*n* = 6575 peaks) which were previously found to characterize healthy pancreas in a study comparing normal to diseased epithelium (Alonso-Curbelo et al. 2021). We assume that these may be associated with critical regulatory events restraining ADM, and thus sought to derive a global GRN mask (applicable to any of the four clusters) with putative regulators binding these regions. This was accomplished by calling motifs using MOODS (Korhonen et al. 2009) with the CisBP motif database (Weirauch et al. 2014) and selecting motif calls meeting significance (*P*-value <1e−10). Putative target genes were then determined with CHIPseeker (Yu et al. 2015), where peaks up to 500 bp upstream and 3000 bp downstream of the transcription start site were mapped to their closest gene. TF–target pairs were merged after filtering for expressed genes, resulting in a GRN mask with dimension equal to the union of targets and TFs (1876), of which 1291 genes are exclusively targets and 585 are TFs (which may be regulated by any other TF). Within this gene-by-gene binary mask, 21.6% of values are nonzero, representing candidate TF–target pairs.

As described, *scKINETICS* applies EM to fit a unique GRN model (*A*) constrained by the binary mask from ATAC-seq data. In highly heterogeneous systems such as this, we learn a separate $A_{c}$ (and therefore separate models of velocity) for each cell type (cluster) c=1…C, dependent in this case on a global mask derived from bulk ATAC-seq and cell-type-specific priors (${\hat{A}}_{c}$ for c=1…C), which are determined from coexpression across cells in a single cluster *c*. Alternatively, when single-cell resolution ATAC-seq is available, cell-type-specific peaks inform a unique TF–target mask per cell type, which is then used to fit $A_{c}$ capturing both unique priors per cell type (${\hat{A}}_{c}$) and the distinct TF–target relationships that exist in each cluster.

To fit the model in this case, we applied *scKINETICS* to each cluster independently, where EM was performed across all target genes for a given cluster until the marginal likelihood plateaus, or up to a maximum of 20 iterations. Using default parameter settings (fixed at $\hat{\sigma }=1$, σ=5), we observe the desired behavior of EM gradually fitting *A* to generate velocities that point toward the center of α and β constraints, while still correlating strongly with the prior values ${\hat{A}}_{ij}$. These values are tunable, and can be updated by the user to obtain a closer fit to the constraints or the prior by decreasing σ or $\hat{\sigma }$ respectively. Each cluster was fed the same binary mask, but unique priors and initializations ${\hat{A}}_{c}$. We then obtained cell-specific velocity estimates as the product of the fitted $A_{c}$ with the expression for cells in cluster *c*, which could then be merged to obtain dynamics for the entire dataset. These vectors may be visualized on 2D embeddings [i.e. Uniform Manifold Approximation and Projection (UMAP) or FDL] using the approach described in La Manno et al. (2018) as a first step toward understanding *scKINETICS* output. We also devised approaches (discussed below) for evaluating their direction in higher dimensions, which more accurately represent their true (genome-wide) orientation.

### 4.2 Velocity analysis

First, we sought to validate whether *scKINETICS* velocity agrees with known biology. Assuming an acinar cell origin, we applied Palantir (Setty et al. 2019) for pseudotime inference to capture the known ADM axis associated with decreasing *Zg16* and increasing *Krt19* expression (Fig. 2A). From a projection of *scKINETICS* velocities on the 2D FDL (Fig. 2B), we found the expected orientation of vectors spanning from acinar-like cells of healthy (uninjured) pancreata toward inflamed duct-like endpoints.

As 2D visualization is insufficient for evaluating directions captured by all genes, we devised a high-dimensional benchmark to compare each cell’s velocity to the pseudotime axis. We reasoned that a given cell’s orientation can be determined by computing the direction that its neighbors point forward (later) in pseudotime. We thus computed “reference” velocities oriented along the ADM axis: for each cell *l*, we choose its neighbor *m* which is the furthest along in pseudotime (i.e. generally most duct-like) and then compute the direction spanned from cell *l* to that neighbor (${\vec{x}}_{m}-{\vec{x}}_{l}$). Across all cells, we ensured these reference directions nearly always point from more acinar-like cells to downstream duct-like cells. To measure the agreement between our predictions and reference directions capturing ADM, we characterized their concordance in direction based on cosine distance for each cell. With this approach, velocity vectors from *scKINETICS* generally pointed parallel to the ADM axis towards duct-like states (<90°), whereas those generated from RNA splicing kinetics such as the field standard *scVelo* (Bergen et al. 2020), and more recently *VeloVAE* (Gu et al. 2022) and *UniTVelo* (Gao et al. 2022), were often more orthogonal to the ADM axis (∼90°; Fig. 2C), and hence were largely incorrect. *scKINETICS* analysis thus suggests a trajectory spanning acinar to duct-like states, as previously described (Kopp et al. 2012), demonstrating that the method is capable of capturing complex trans-differentiation events, even when standard velocity approaches fail.

### 4.3 Robustness analysis

**Figure 3.**
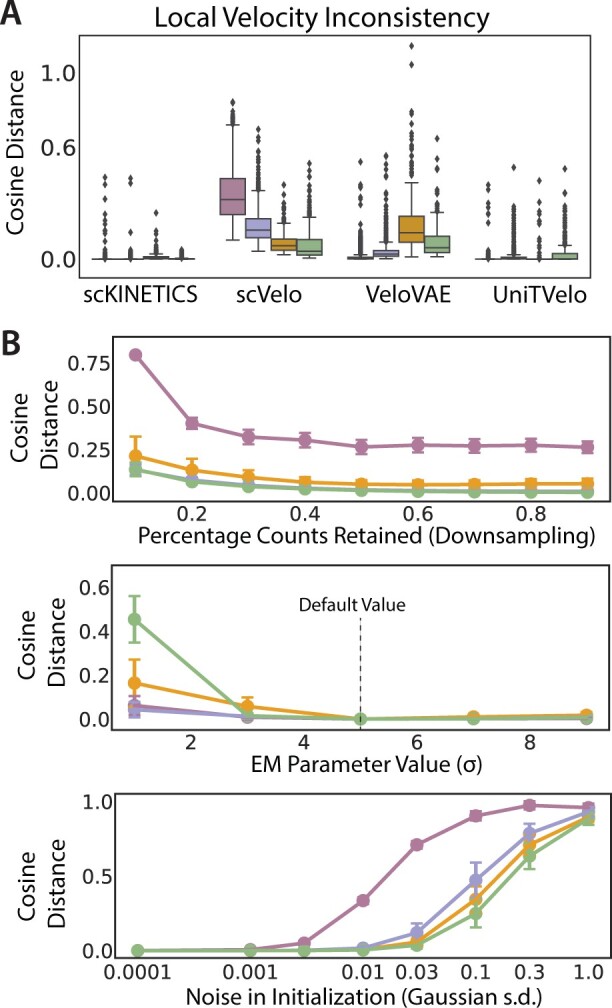
Robustness analysis in local consistency and sensitivity to perturbation and noise. (A) *scKINETICS* outperforms competing methods *scVelo*, *VeloVAE*, and *UniTVelo* in local velocity vector consistency between *k* = 30 nearest neighbors, indicating a closer match between the directions of velocities among neighboring cells. Colors correspond to Phenograph clusters from Fig. 2. (B) Sensitivity of *scKINETICS* to technical features of scRNA-seq, user-defined parameters, and choice of prior in initialization. Cosine distances are between bootstrapped and original velocity, such that lower values imply a closer match to the original vectors. Random downsampling of the raw scRNA-seq count matrix shows robustness against technical noise such as dropout. Varying values of the EM parameter σ around the default value demonstrate robustness to user input. Random noise added to the Coexpression Prior $\hat{A}$ shows robustness to noise in prior and initiation values.

While the vectors inferred from *scKINETICS* appear to capture an axis of pancreatic regeneration on a per-cell basis, previous authors have noted that longer range dynamics (i.e. spanning initiating to terminal states) may be poorly captured by velocity approaches, which may produce highly inconsistent or noisy results. To enable inference of longitudinal dynamics, *post hoc* methods such as *CellRank* (Lange et al. 2022) integrate per-cell velocities with manifold-level information (e.g. cell–cell similarity), improving the prediction of trajectories from origins to endpoints. We suspected that *scKINETICS* may directly mitigate some of the issues with velocities by leveraging similar information up-front during velocity inference through the “**Manifold Constraint” (3.2.1.2)**. To evaluate this, we first sought to assess the level of local inconsistency between velocities of neighboring cells, which are expected to be low given that neighbors tend to have similar expression profiles. Using the kNN graph, we quantified “inconsistency” as the cosine distance between a cell’s velocity vector and the vectors of its *k* = 30 neighbors. *scKINETICS* generated highly concordant velocities, with an average cosine distance of 0.0029 (4.351°) across all cells. In contrast, competing algorithms generated more inconsistent vectors in all clusters, with an average cosine distance of 0.2419 for *scVelo*, 0.0449 for *VeloVAE*, and 0.0077 for *UniTVelo*. These methods even predicted orthogonal vectors (cosine distance 1) between neighboring cells in some cases (Fig. 3A).

As splicing-based velocity estimation may be highly sensitive to technical fluctuations in gene expression (e.g. outliers which can impact the dynamics for a given gene), one source of such inconsistencies may be noise in the expression profiles of cells. We designed a robustness experiment to determine whether *scKINETICS* provides stable estimates regardless of these technical factors, focusing specifically on the highly influential transcript capture rate. From the raw scRNA-seq count matrix, we randomly downsampled the counts of transcripts to several different percentages of the total original counts (Fig. 3B), simulating the impact of drop-out and poor gene capture across the transcriptome. In 15 trials at each downsampling proportion, we fit the *scKINETICS* model with the altered count matrix, holding constant the accessible peaks (defining the GRN mask) and any tunable parameters for EM. The deviation of the resultant “perturbed” velocity from the original is quantified by cosine distance. Performing this test on the pancreatic regeneration dataset, we observed minimal effects from the perturbation.

In additional experiments varying user-defined parameter choice (σ, described in Section 3.2.1.2) or choice of initialization and prior ($\hat{A}$, described in Section 3.2.1.1) holding all other inputs constant, we observed a similar degree of robustness within reasonable windows. We observed nearly perfect recovery of velocities over a wide range of initiating σ values, and modest deviation (cosine distance < 0.6) even with extreme values of σ (Fig. 3B). To test the impact of the prior and initialization, we added successive amounts of Gaussian-distributed noise to nonzero elements of $\hat{A}$. The resultant vectors showed relatively consistent alignment to the original vectors inferred without noise, with elevated deviations under extreme noise addition substantially exceeding the scope of true $\hat{A}$ values, which are on the order of $10^{-4}$ on average (Fig. 3B). These experiments affirm the utility of our choice of default parameters (σ=5), initialization, and prior in recovering velocities pointing along ADM, but also suggest that the model may recover useful biology under deviation from these values.

Lastly, to support the accuracy of inferred *A* matrices (for which we lack ground truth), we performed a simulation study with data generated from the model with added noise. Using a fitted *A* matrix and a randomly chosen start cell $\vec{x}(0)$, we simulated expression profiles at known time points *t* by evaluating $\vec{x}(t)=e^{At}\vec{x}(0)$, then added Gaussian noise centered at 0 (SD = 5). In 15 such experiments from distinct start cells, the inferred *A* was correlated to the true *A* matrix to an average of r=.665 considering only nonzero elements, supporting the ability of *scKINETICS* to build an accurate GRN.

### 4.4 TF activity analysis

Our analysis of regeneration data demonstrates the utility of *scKINETICS* velocities to nominate trajectories and their dynamics through time. As the ADM trajectories we observed consist of multiple distinct clusters, we asked whether *scKINETICS* might shed light on the heterogeneity of duct-like states derived from a single acinar population. We reasoned that differential TF activity may be what enables the diversity in trajectories observed from a relatively homogeneous origin, and thus sought to use the TF inference component of *scKINETICS* to quantify the regulatory activity driving distinct cell populations.

**Figure 4.**
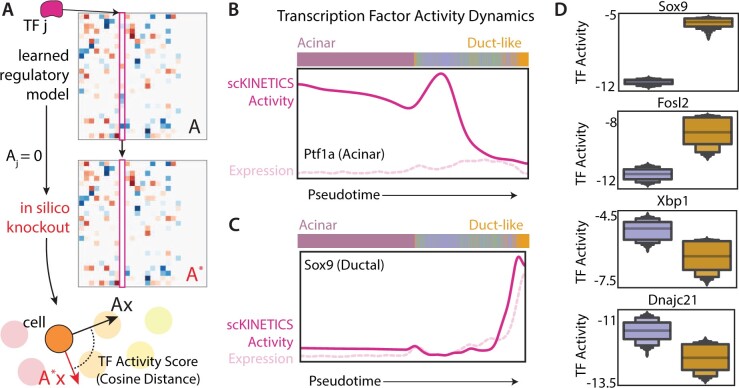
*scKINETICS* enables TF-wide *in silico* perturbation screening. (A) Schematic of the *in silico* TF knockout experiment. From each cluster’s *A* matrix (GRN), a column representing a TF can be zero-ed out to mimic the loss of impact on target expression rates caused by the inactivation of that TF. A “perturbed” velocity (red) is calculated with the altered $A^{*}$ matrix, and its cosine distance from the original velocity vectors defines the TF’s activity score per cell. (B and C) TF activity dynamics of acinar marker *Ptf1a* (B) and ductal marker *Sox9* (C). Solid lines denote TF perturbation score obtained from (A), dotted lines denote TF gene expression; both are standard scaled and smoothed for visualization, and cells are ordered along pseudotime. The expected decline in *Ptf1a* activity across pseudotime is observed despite its low expression throughout, and *Sox9* activity and expression are highly concordant along ADM. (D) Activity scores for cells in ductal clusters for TFs *Sox9*, *Fosl2*, *Xbp1*, and *Dnajc21*. Within a distribution, the mean is denoted by the gray line and each box represents a quantile.

A key feature of *scKINETICS* is that it can be used for a TF-wide *in silico* perturbation screen of regulators with strong downstream impacts. Our expressive, TF-centric model of transcriptional dynamics is a natural framework for simulating the functional consequences of a particular regulatory event. Assuming that the learned GRN can faithfully capture future states, alterations to the regulatory model are expected to modify these predictions in proportion to the importance of the perturbed TF. With this in mind, we designed a score that tracks the activity of each TF across individual cells based on an *in silico* TF knockout experiment. A column of *A* ($A_{j}$, representing the regulatory weights for TF *j* across all targets) can be set to zero to define a perturbed matrix $A^{*}$. This emulates a knockout experiment, where the TF *j* can no longer impact any target’s expression, as the regulatory weight for each $A_{ij}$ is fixed at zero for all *i*. Velocities computed under this perturbed matrix as ${\vec{x}}^{\prime }(t)=A^{*}\vec{x}$ represent the predicted dynamics if TF *j* were inactive. We then assume that a large change in the perturbed velocity of a given cell relative to its original (unperturbed) velocity indicates that TF *j* has an equally large impact on the predicted dynamics of that population. Hence, the activity score for TF *j* is quantified by the cosine distance between the original ($A\vec{x}$) and altered velocity vectors ($A^{*}\vec{x}$) for each cell (Fig. 4A).

This approach can be used to assess the activity of any regulator in any population, allowing us to screen the activity of all 585 TFs in each of the 5501 single cells analyzed. Our first goal was to determine whether the screen can nominate known regulators of acinar or duct-like states. Importantly, as the score combines both epigenetic data (dictating the targets or nonzero values, in the regulatory model for TF *j*) and expression data (dictating the magnitude of nonzero values), our activity scores may capture heterogeneity in TF activity which is undetectable by one modality alone. In general, due to their low expression, TFs are often poorly captured in scRNA-seq data, suggesting that the incorporation of additional data (i.e. knowledge of candidate target genes from epigenetic data) may improve the detection of their activities across cells. We thus sought to establish whether our activity score might highlight TFs that would not stand out from expression data alone.

A prime example in our dataset was *Ptf1a*, a canonical acinar TF (Kawaguchi et al. 2002) which has surprisingly low expression in all cells analyzed, including those with clear acinar phenotypes (Fig. 4B). Indeed, *Ptf1a* activity inferred by *scKINETICS* is elevated across the acinar and early transitioning cell populations, with declining regulatory strength along ADM in concordance with the literature. On the other hand, *Sox9*, a canonical ductal TF, shows matched expression and regulatory activity as it increases at duct-like endpoints (Fig. 4C). Thus, we find that the activity score often correlates with expression (as expected) in cases where TFs are robustly expressed, but may still capture critical TF activity in the absence of substantial expression through the knowledge of TF–target interactions.

The TF activity score can be seen as a powerful hypothesis-generating tool to predict regulators driving population-specific dynamics. As we observed distinct clusters of duct-like cells emerging through ADM, we used *scKINETICS* to speculate on differential regulators driving these putative branches. Using pairwise t-tests on the activity scores for each TF across cells of each duct-like cluster, we identified factors with strongly differential activity across populations. Among the elevated TFs in one duct-like cluster are *Fosl2* and other AP1 family members (Fig. 4D), which have known roles in driving pancreatic tumorigenesis (Vallejo et al. 2017; Alonso-Curbelo et al. 2021). Interestingly, while *Sox9* activity increases along ADM in general (see Fig. 4C), we predict that it is induced much more robustly in one cluster relative to another (Fig. 4D). The other duct-like cluster is instead defined by high *Xbp1* activity, a TF that is responsive to ER stress and promotes pancreas regeneration (Hess et al. 2011). Likewise, heat shock regulator *Dnajc21* shows high regulatory activity in this branch (Fig. 4D), which is consistent with rising expression of heat shock genes (e.g. *Hspb1*) observed in this population. Thus, the TF activity score proposes novel regulators of distinct metaplastic populations, many of which have known roles in pancreas biology, or may comprise consistent regulatory programs relating to increased cellular stress, but all of which agree with expectations based on the expression patterns of target genes that differentiate these populations.

## 5 Discussion

*scKINETICS* is an algorithm for the joint inference of transcriptomic velocity and regulatory events based on a dynamic model of TF–target interactions. Our approach is based on a custom EM algorithm and incorporates several unique features that drive performance.

Previous work demonstrated that incorporating manifold-level information *post hoc* can improve velocity directions, which are inherently noisy (Lange et al. 2022). *scKINETICS* is, to our knowledge, the first algorithm to leverage the generous information that the phenotypic manifold provides on future states up-front during velocity computation. We further improve inference by incorporating additional biologically motivated priors, including gene–gene coexpression patterns, which have previously been shown to dramatically improve inference in complementary settings (Prabhakaran et al. 2016; Bachireddy et al. 2021). These features likely contribute to its superior consistency over previous methods, which have implications for the integration of velocities across the manifold in trajectory inference.

In a pancreatic regeneration dataset, we demonstrated improved accuracy compared with standard velocity algorithms based on RNA splicing (*scVelo*, *UniTVelo*, *VeloVAE*). *scKINETICS* is currently more widely applicable than emerging velocity approaches based on epigenetic information, which require concurrently assayed single-cell resolution measurements (*MultiVelo*). While incorporation of single-cell-level accessibility information would likely be valuable in velocity inference, the lack of paired per-cell accessibility data in the pancreatitis setting made it impossible for us to compare our approach to *MultiVelo* directly. Even so, *scKINETICS* is unique in incorporating gene-level mechanistic information in the inference procedure: whereas *MultiVelo* models accessibility at peaks without knowledge of associated regulatory proteins, *scKINETICS* models transcriptional change as a function of regulator activity. This provides a conceptual advantage, in that velocity inference depends on both peak accessibility and regulator expression, a combination that has proven beneficial in prior work (Argelaguet et al. 2022).

Furthermore, unlike multiome-based approaches, this interpretable modeling scheme allows us to generate mechanistic hypotheses about key regulators driving phenotypic change. We developed an *in silico* perturbation approach which captures cell-level TF binding that accounts for both TF and target expression patterns. The approach can be expanded to TF-wide screens that can recover known regulator dynamics despite limited prior knowledge (Fig. 4). A recent analogous approach, CellOracle (Kamimoto et al. 2023), applies *in silico* regulator perturbation based on a GRN model of current target gene expression (i.e. leveraging TF expression in the same cell), whereas *scKINETICS* models future dynamics in expression represented by velocities. We perform inference to jointly learn a GRN along with these cell-state dynamics, as opposed to CellOracle which derives information on cell-state dynamics dependent on a learned GRN. Indeed, we show that *scKINETICS*-derived TF activity scores may more faithfully recover known regulatory dynamics in pancreatitis than gene expression alone, and further demonstrate insights that may be generated on the differential regulators of distinct cell states. Our approach holds promise for nominating important regulators of cell states across biological domains for prioritizing experimental perturbation.

Currently, *scKINETICS* may be limited by the inference of TF–target pairs through motif calling and distance-based target association, both of which can be noisy and discard critical genes. While we show that our model can recover important regulators despite these features, future work may improve regulatory network inference by incorporating additional information such as Hi-C or perturbation data. In addition, our modeling approach allows for sensible differences between disparate cell types and is designed for flexibility to diverse data types, including those (bulk ATAC-seq) with limited cell-type-specific information. However, we expect substantial gains in accuracy and interpretability from future mechanistic approaches leveraging high-resolution single-cell measurements, particularly those with multiome data. These have already begun to emerge in domains related to GRN inference (Argelaguet et al. 2022; Fleck et al. 2022) or velocity (Li et al. 2023), and have yet to be applied in a joint approach. Future work may also integrate *scKINETICS* vectors across the manifold using approaches such as *CellRank*, which can infer longitudinal dynamics from these local velocity estimates.

## 6 Conclusion

*scKINETICS* can generate robust, interpretable insights into phenotypic dynamics and their regulatory underpinnings. Application of these approaches to diverse biological settings holds promise for uncovering key regulators underlying tissue heterogeneity.

## Data Availability

All data used in this article are available from public sources, as detailed above in Section 4.1.
